# Associations between Coping Strategies and Cyberhate Involvement: Evidence from Adolescents across Three World Regions

**DOI:** 10.3390/ijerph19116749

**Published:** 2022-05-31

**Authors:** Sebastian Wachs, Juan Manuel Machimbarrena, Michelle F. Wright, Manuel Gámez-Guadix, Soeun Yang, Ruthaychonnee Sittichai, Ritu Singh, Ramakrishna Biswal, Katerina Flora, Vassiliki Daskalou, Evdoxia Maziridou, Jun Sung Hong, Norman Krause

**Affiliations:** 1 Department of Educational Studies, University of Potsdam, 14476 Potsdam, Germany; normankrause@uni-potsdam.de; 2 National Anti-Bullying Research and Resource Centre, Dublin City University, D09 AW21 Dublin, Ireland; mwrigh20@depaul.edu; 3 Faculty of Psychology, University of the Basque Country, 20018 Donostia, Spain; juanmanuel.machimbarrena@ehu.eus; 4 Department of Psychology, DePaul University, Chicago, IL 60604, USA; 5 Department of Biological and Health Psychology, Autonomous University of Madrid, 28049 Madrid, Spain; manuel.gamez@uam.es; 6 Center for Digital Humanities & Computational Social Sciences, Korea Advanced Institute of Science and Technology, Daejeon 34141, Korea; soeun0228@kaist.ac.kr; 7 Kids and Youth Development Research Center, Research Center for Educational Innovations and Teaching and Learning Excellence, Faculty of Humanities and Social Sciences, Prince of Songkla University, Muang, Pattani 94000, Thailand; ruthaychonnee.s@psu.ac.th; 8 Department of Human Development and Family Studies, G.B. Pant University of Agriculture and Technology, Pantnagar 263145, India; ritu.singh07@gmail.com; 9 Department of Humanities and Social Sciences, National Institute of Technology, Rourkela 769008, India; rkbpsych@gmail.com; 10 Department of Psychology, Neapolis University Pafos, Pafos 8042, Cyprus; k.flora@nup.ac.cy; 11 Department of Psychology, Aristotle University of Thessaloniki, 54124 Thessaloniki, Greece; vassilikida@yahoo.gr (V.D.); evamazirid@gmail.com (E.M.); 12 School of Social Work, Wayne State University, Detroit, MI 48202, USA; fl4684@wayne.edu

**Keywords:** cyberhate, hate speech, coping strategies, cross-national, counter-speech

## Abstract

Cyberhate represents a risk to adolescents’ development and peaceful coexistence in democratic societies. Yet, not much is known about the relationship between adolescents’ ability to cope with cyberhate and their cyberhate involvement. To fill current gaps in the literature and inform the development of media education programs, the present study investigated various coping strategies in a hypothetical cyberhate scenario as correlates for being cyberhate victims, perpetrators, and both victim–perpetrators. The sample consisted of 6829 adolescents aged 12–18 years old (*M_age_* = 14.93, *SD* = 1.64; girls: 50.4%, boys: 48.9%, and 0.7% did not indicate their gender) from Asia, Europe, and North America. Results showed that adolescents who endorsed distal advice or endorsed technical coping showed a lower likelihood to be victims, perpetrators, or victim–perpetrators. In contrast, if adolescents felt helpless or endorsed retaliation to cope with cyberhate, they showed higher odds of being involved in cyberhate as victims, perpetrators, or victim–perpetrators. Finally, adolescents who endorsed close support as a coping strategy showed a lower likelihood to be victim–perpetrators, and adolescents who endorsed assertive coping showed higher odds of being victims. In conclusion, the results confirm the importance of addressing adolescents’ ability to deal with cyberhate to develop more tailored prevention approaches. More specifically, such initiatives should focus on adolescents who feel helpless or feel inclined to retaliate. In addition, adolescents should be educated to practice distal advice and technical coping when experiencing cyberhate. Implications for the design and instruction of evidence-based cyberhate prevention (e.g., online educational games, virtual learning environments) will be discussed.

## 1. Introduction

Currently, a major challenge for democratic societies is the spread of fake news, conspiracy narratives, and cyberhate [1,2]. Adolescents are the age group that is more active online than any other [3]. It is, therefore, not surprising that adolescents are increasingly exposed to cyberhate [4,5,6]. This is concerning because adolescence is a formative period for political socialization processes which are also influenced by online experiences and the use of information and communication technologies [7]. Moreover, exposure to cyberhate is associated with negative outcomes, including increases in offline hate crimes, higher levels of outgroup prejudices, lower well-being, and aggressive behavior [6,8,9,10,11]. Hence, it is crucial to understand how adolescents can cope with cyberhate. Although many studies investigated how adolescents deal with related online risks (e.g., cyberbullying), little research attention has been given to adolescents’ coping strategies for dealing with cyberhate. To this end, the present study examines the associations between cyberhate-specific coping strategies and adolescents’ likelihood of cyberhate involvement. The findings can be used to inform the development of media education prevention programs and the development of online learning environments with the aim of improving adolescents’ ability to cope with cyberhate, thus reducing their likelihood of becoming involved in cyberhate.

### 1.1. Adolescents’ Coping with Cyberhate and Involvement in Cyberhate

Cyberhate (also known as hate speech) is usually defined as the sharing, creating, or forwarding of offensive, mean, or threatening, posts, comments, text messages, videos, and pictures via information and communication technologies targeting a group or person because of their gender, sexual orientation, disability, race, ethnicity, nationality, or religion [1]. Cyberhate shows conceptual and empirical overlaps with cyberbullying. Cyberbullying is often described as a hostile and repeated negative behavior against people who cannot easily defend themselves and is carried out among people of relatively stable social groups. Cyberhate can, however, also be carried out as a single act among strangers. While cyberbullying is targeting often people based on individual characteristics, cyberhate is necessarily directed against people because of actual or assigned memberships to social groups [12,13,14].

Being the target of or being exposed to cyberhate is a stressful experience and can evoke negative emotions [6,15,16]. The ability to manage stressful events through cognitive, emotional, and behavioral efforts is defined as coping [17]. Based on the Transactional Model of Stress and Coping [17], Frydenberg [18] proposed a coping typology for adolescents consisting of three coping styles:

1. *Reference to others* includes strategies to engage with others to cope, such as seeking emotional support from close friends or family members (*close support*) or asking for informational and instructive advice from teachers or other professionals (*distal advice*).
2. The *productive coping style* comprises strategies to deal with the stressor and includes defending oneself and confronting the cyberhate perpetrator without causing any harm (*assertiveness*). It also includes strategies such as blocking the cyberhate perpetrator to protect personal information online to increase protection (*technical coping*). Reference to others and productive coping can both be understood as functional coping styles that are used with the intention or the belief in the ability to manage or change the problem causing the distress.
3. The *non-productive coping style* includes strategies such as coping through counter-aggressions (*retaliation*) or the belief that one is not capable of dealing with cyberhate incidents (*helplessness*). The non-productive coping style is considered dysfunctional and is often employed with the belief that a person cannot change or stop the stressor.

Research investigating how adolescents cope with cyberhate is scarce. One study found that adolescents’ most frequently used coping actions were ignoring cyberhate when exposed to it, which was followed by reporting it to online service providers, speaking to a friend about it, blocking the person who shared the cyberhate, telling a parent or another adult about it, replying publicly to the perpetrator, informing a teacher or other professional, and reporting the behavior to the police [19]. More recently, current quantitative research based on a multidimensional scale for measuring coping with cyberhate revealed that adolescents used technical coping, assertiveness, and close support most frequently, followed by helplessness/self-blame, retaliation, and distal advice [20,21]. Similarly, Krause et al. [6] found based on qualitative interviews that German adolescents coped with experiences of hate speech in schools by referring to social support, avoidance, active ignoring, and counter-speech.

Adolescents are involved in cyberhate through several roles. They can observe cyberhate (witnesser), be targeted by cyberhate material (victim), post, forward, or share harmful or hostile cyberhate material (perpetrator), and be both victim and perpetrator (victim–perpetrator [22,23]). While initial research has increased our understanding of how adolescents cope with cyberhate, there is nearly no information on how coping strategies with cyberhate are related to adolescents’ involvement in cyberhate as victims, perpetrators, or both. Given the sparse research on coping with cyberhate, literature on adolescents’ strategies to cope with offline and online aggression will be reviewed. Prior research on associations between coping strategies and involvement in online and offline aggression is mixed and difficult to compare because different typologies of coping styles and strategies have been used in the literature. In addition, coping strategies are highly dependent and nuanced by several other variables, such as the nature of the stressor, context, age, and severity [24], which might further add to the contradictory research findings in the literature.

#### 1.1.1. Reference to Others

The reference to others’ coping styles is one of the most highly regarded responses to cybervictimization by adolescents and professionals [25,26,27]. There is, however, some contention within the literature concerning whether the reference to others is effective in coping with traditional and cyber victimization. Some research demonstrated that asking others for advice was negatively correlated with cyberbullying victimization [28,29], while other research did not indicate an association between using social support and being victims of cyberbullying [30,31]. Yet other research distinguished between the resource of support and found a negative relationship between seeking help from teachers and being victims of cyberbullying but not from peers or family [32]. There is also another line of research that reported a negative association between seeking help from friends and being victims of cyberbullying but not from parents or teachers [33]. Nonetheless, other research showed a negative correlation between seeking support from peers or adults and being at risk for traditional victimization [34]. Research on the use of reference to others as a coping style endorsed by victims of cyberhate is scarce with initial research showing a negative relationship between coping by reference to others and cyberhate victimization [20]. Investigations on the association between coping and aggressive behavior generally showed that functional coping strategies are negatively correlated with perpetrating aggression [35]. More specifically, regarding reference to others, some research showed that lower levels of social support were linked to higher levels of traditional aggression [36,37,38] and cyber aggression [39].

Adolescents who have strong social support might be less likely to become victims than their more isolated peers or those who have difficulties with social interactions because positive interactions with peers and family protect against victimization [40,41,42]. In the same line, social support might help to reduce stress and frustration and, thus, makes it less likely that adolescents engage in reactive aggression [37,43] and become a perpetrator of cyberhate themselves. Hence, it can be assumed that reference to others is negatively related to being victims, perpetrators, and victim–perpetrators of cyberhate.

#### 1.1.2. Productive Coping

It has been emphasized by several researchers that adolescents most frequently use a productive coping style to deal with varying online risks [20,24,32,44,45,46,47]. Assertiveness (i.e., confronting the aggressor, telling the aggressor to stop) has received mixed results in the literature, as it is positively related to a higher risk of cyberbullying victimization [28] and a lower risk of cybergrooming victimization [48]. Other researchers, however, have found a negative association with the likelihood of experiencing cyberbullying victimization [39], whereas other research did not find any correlation with cyberbullying victimization [49]. Regarding cyberbullying perpetration, one study that investigated potential associations between assertiveness and cyberbullying perpetration suggests no significant relationship [39], and another found a negative relationship between problem-focused coping in general and cyberhate perpetration [47].

Another productive coping strategy, namely technical coping (i.e., blocking the aggressor, paying attention to security settings), has also revealed mixed findings relating to preventing (future) victimization. While Wachs et al. [48] found that using technical coping was positively correlated with being victims of cybergrooming, other scholars found a negative relationship between utilizing technical coping and being victims of cyberbullying [32]; yet other researchers did not find a correlation between technical coping and cyberbullying victimization or perpetration [39].

Productive coping might reduce adolescents’ risk for cyberhate victimization through the concept of cognitive appraisal and positive thinking. Cognitive appraisal is what a person does to evaluate whether a particular encounter is relevant to his or her well-being. Since the introduction of the principle of appraisal by Lazarus and Folkman [50], the benefit of positive thinking when coping with stress has been acknowledged [51]. Positive thinking might allow adolescents to interpret stressful situations in ways that are conducive to growth and success and, therefore, would prevent adolescents from participating or engaging in any further cyberhate encounters, either by blocking the attacker or by being assertive toward the perpetrator. In addition, it is well known that productive coping strategies allow people to adjust better to stressful situations [50]. More specifically, research has shown that productive coping mitigates the negative consequences of traditional and cybervictimization on psychological functioning [52,53,54], increases efficacy when dealing with stressful situations, and therefore reduces reactive aggression.

#### 1.1.3. Non-Productive Coping

In general, non-productive coping, such as helplessness and retaliation, was less frequently recommended by students for victims compared with reference to others or productive coping [55]. Prior research has revealed a positive relationship between retaliation and being victims of cyberhate [20] as well as traditional or cyberbullying [30,31,46,56]. Regarding perpetrators, there is some evidence that several non-productive strategies, such as helplessness, self-blame, and retaliation, were positively correlated with offline and online aggression [31,35,47,56,57].

Non-productive styles are particularly worrying coping styles, as strategies such as helplessness, self-blame, or revenge were linked to a higher level of depressive symptoms and suicidal ideation, whereas reference to others and productive coping were related to lower levels of depressive symptoms [55,58,59]. Furthermore, while retaliation can prolong a vicious circle of violence and implicate the individual further into cyberhate with a dual role (i.e., victim and perpetrator [60]), helplessness (i.e., rumination and self-blame) could lead to maladaptive schemas and the chronification of victimization [61]. Therefore, it can be assumed that non-productive coping is positively associated with being victims, perpetrators, and victim–perpetrators of cyberhate.

### 1.2. The Present Study

The present study sought to examine the associations between varying coping styles (i.e., reference to others, productive coping, and non-productive coping) and adolescents’ cyberhate involvement risk. First, it was hypothesized that adolescents who endorsed close support and distal advice (*reference to others*) will show lower odds of being victims, perpetrators, and victim–perpetrators of cyberhate compared with adolescents who were not involved in cyberhate (**H1**). Second, it was hypothesized that adolescents who endorsed assertive and technical coping (*productive coping*) will show lower odds of being victims, perpetrators, and victim–perpetrators of cyberhate compared with those who were not involved in cyberhate (**H2**). Third, it was hypothesized that adolescents who endorsed helplessness and retaliation coping (*non-productive coping*) will show higher odds of being victims, perpetrators, and victim–perpetrators of cyberhate compared with those who were not involved in cyberhate (**H3**).

## 2. Materials and Methods

### 2.1. Participants

The sample included 6829 adolescents aged 12–18 years old (*M_age_* = 14.93; *SD* = 1.64; girls: 50.4%, boys: 48.9, 0.7% did not indicate their gender) from three world regions: Asia (India, South Korea, Thailand), Europe (Cyprus, Greece, Germany, Spain), and North America (USA). By country, the study sample included 847 American participants (12–18 years; *M_age_* = 14.79; *SD* = 1.80; girls: 49.2%, boys: 47.9%, 2.8% did not indicate their gender), 221 Cypriot participants (12–18 years; *M_age_* = 14.49; *SD* = 1.48; girls: 67.4%, boys: 32.7, 0.9% did not indicate their gender), 1480 German participants (12–17 years; *M_age_* = 14.21; *SD* = 1.23; girls: 50.3%, boys: 49.7%), 670 Greek participants (15–18 years; *M_age_* = 16.49; *SD* = 1.12; girls: 52.8%, boys: 45.7%, 1.5% did not indicate their gender), 1121 Indian participants (13–18 years; *M_age_* = 15.37; *SD* = 1.48; girls: 45%, boys: 55%), 756 South Korean participants (12–17 years; *M_age_* = 14.73; *SD* = 1.23; girls: 49.7%, boys: 50.1%, 0.1% did not indicate their gender), 1018 Spanish participants (12–18 years; *M_age_* = 14.29; *SD* = 1.64; girls: 51.6%, boys: 48.2%, 0.2% did not indicate their gender), and 716 Thai participants (13–18 years; *M_age_* = 15.68; *SD* = 1.70; girls: 52.1%, boys: 46.6, 1.3% did not indicate their gender). Table 1 shows the distribution of participants by age, sex, and country of origin.

### 2.2. Measures

**Cyberhate Involvement**. The instrument for measuring cyberhate involvement consisted of a definition of cyberhate and two single items to measure cyberhate involvement. The given definition was “Online hate describes the usage of information and communication technologies (e.g., WhatsApp, Facebook, Instagram, Twitter) to offend and hurt somebody because of his or her race, gender, ethnic group, nationality, disability, sexual orientation, or religion. It can be either targeted directly at a person or group or generally shared online. Online hate can be offensive, mean, or threatening and can be expressed through degrading writings or speech online such as posts, comments, text messages, videos or pictures.” The items to measure cyberhate involvement were adopted by Hawdon et al. [62]. One item was used to measure cyberhate victimization: “How often did it happen in the past 12 months that you have personally been the target of hateful or degrading writings or speech online because of your sex, religious affiliation, race, or sexual orientation?”. For cyberhate perpetration, the following item was used: “How often did it happen in the past 12 months that you have posted hateful or degrading writings or speech online, which inappropriately attacks certain groups of people or individuals based on their sex, religious affiliation, race, or sexual orientation?”. Both items were answered on a five-point scale: “never” (0), “very rarely” (1), occasionally (2), frequently (3), and “very frequently” (4).

To investigate distinct associations among pure victims, pure perpetrators, and victim–perpetrators of cyberhate, the cyberhate victimization, and perpetration variables were recoded into one multinomial variable with four distinct groups. Pure cyberhate victims scored only higher than “never” on the cyberhate victimization item, pure cyberhate perpetrators reported only higher than “never” on the cyberhate perpetration item, cyberhate victim–perpetrators scored higher than “never” on both cyberhate victimization and perpetration items, and non-involved (i.e., adolescents who were not involved in cyberhate) reported “never” on both variables. We decided to use a categorical analyses approach to embrace the skewed distributions of the cyberhate variables and allow for the comparison of distinct associations between coping strategies and being a cyberhate victim, perpetrator, and both. Consequently, we accepted the loss of statistical power but avoided biased parameter estimates due to non-normal deviated outcomes [63,64].

**Coping Strategies**. Coping strategies were measured by a validated instrument to measure adolescents’ coping strategies with cyberhate [20]. Participants rated their endorsement of three coping styles with six subscales total. The first coping style is *Reference to others*, and it includes two subscales: Distal advice (3 items, e.g., “… go to the police”; α = 0.81) and Close support (4 items, e.g., “… spend time with my friends to take my mind off it”, α = 0.84). *Productive coping* is the second coping style and includes two subscales: Assertiveness (4 items, e.g., “… tell the person to stop it”; α = 0.88) and Technical coping (3 items, e.g., “… block that person so that he/she cannot contact me anymore”; α = 0.83). *Non-productive coping* is the third coping style and includes two subscales: Helplessness/Self-blame (3 items, e.g., “… not know what to do”; α = 0.76) and Retaliation (3 items, e.g., “… do it back”; α = 0.77). All items were rated on a scale: “definitely not” (0), “probably not” (1), “probably” (2), and “definitely” (3). Supplementary Table S1 provides coefficient alphas by country.

**Control variables**. Adolescents’ age, sex (male versus female), and country of origin were used as control variables, as there is some initial research that showed differences in demographic variables concerning cyberhate involvement [4,9,22,65].

### 2.3. Procedure

Approval to conduct this research was received from the Institutional Review Boards of the associated researchers’ universities, and the Helsinki ethics protocol was followed for this study [66]. Data for this project were collected by first contacting school principals via emails or calls to discuss the aims of the study. Upon securing approval from the school principals, classroom announcements about the study were made in the participating schools. Parental permission slips were sent home with adolescents to acquire consent for participation from the parent(s)/guardian(s). The response rate at the student level among all participating countries was between 71% and 85%. Adolescents were informed that their participation is voluntary and that they could stop taking part in the study whenever they want to or leave certain questions out if they were not comfortable answering the questions. Data were collected during regular school hours.

The research team followed the recommended process to translate the survey between various languages. This helped ensure that students in different countries were responding to the same set of questions and that the respective results were therefore comparable. The process included first translating the original instruments into the target language and then translating them back by someone who had not seen the original questionnaires. Finally, the new translation was compared to the original instrument to ensure consistency [67]. All translated versions can be requested from the first author.

### 2.4. Data Analyses

Descriptive statistics and correlations were computed for all main study variables. Subsequently, the polynomial cyberhate involvement variable was used in a multinomial logistic regression analysis as the dependent variable to investigate the prediction of cyberhate involvement by participants’ endorsement of several coping strategies while controlling for participants’ sex, age, and country of origin. The correlation matrix was evaluated to examine multicollinearity before conducting the multinomial logistic regression analysis (see Table 2).

The results indicated that all coping strategies were suitable for consideration, as independent variables in one multinomial regression analysis did not detect any high correlations (>0.70). Missing values ranged between 1.2% (cyberhate victimization) and 3.5% (close support). The Little’s MCAR test revealed that the data were missing completely at random (χ^2^ = 131.85 *df =* 111; *p* = 0.086), suggesting a pairwise or listwise deletion of missing data does not lead to biased parameters and standard errors [68]. All analyses were conducted using IBM SPSS Statistics software version 26 (SPSS Inc., Chicago, IL, USA) for Mac. 

## 3. Results

Descriptive statistics and bivariate correlations of all main study variables are included in Table 2. Supplementary Table S2 provides descriptive statistics and correlations among the main study variables by country. Overall, 11.4% (*n* = 780) were pure victims, 6% (*n* = 409) were pure perpetrators, 8% (*n* = 548) were victim–perpetrators, 73.2% (*n* = 4998) were non-involved, and 1.4% (*n* = 94) could not be classified due to missing values. Supplementary Table S3 provides a breakdown of cyberhate involvement by country.

The multinomial regression analyses revealed several statistically significant associations among the six coping strategies, namely distal advice, close support, assertiveness, technical coping, helplessness, revenge, and being victims, perpetrators, or victim–perpetrators of cyberhate while controlling for participants’ age, sex, and country of origin (see Table 3). The model was significant, Log-likelihood (null) = 6970.33; Log-likelihood (full) = 6101.35; Likelihood Ratio Chi-Square test = 868.97, *df* = 45, *p* < 0.001, Nagelkerke’s *R*^2^ = 0.155.

**Reference to others**: Distal advice decreased the likelihood of classifying a participant as being a cyberhate victim (OR = 0.687, CI_95%_ [0.578–0.817]), perpetrator (OR = 0.541, CI_95%_ [0.422–0.692]), and victim–perpetrator (OR = 0.820, CI_95%_ [0.726–0.925]); i.e., if participants endorsed distal advice, they were less likely to be cyberhate victims, perpetrators, or both than non-involved participants. Close support predicted lower odds of being a cyberhate victim–perpetrator (OR = 0.669, CI_95%_ [0.533–0.840]); i.e., if participants endorsed close support, they were less likely to be victim–perpetrators than non-involved participants.

**Productive coping**: Assertiveness predicted higher odds of being a cyberhate victim (OR = 1.23, CI_95%_ [1.11–1.51]); i.e., if participants endorsed assertive coping with cyberhate, they were more likely to be victims than non-involved participants. Technical coping predicted lower likelihoods of being a cyberhate victim (OR = 0.755, CI_95%_ [0.622–0.918]), perpetrator (OR = 0.533, CI_95%_ [0.415–0.685]), and victim–perpetrator (OR = 0.419 CI_95%_ [0.337–0.521]); i.e., if participants endorsed technical strategies to cope with cyberhate, they were less likely to be victims, perpetrators, or victim–perpetrators than non-involved participants.

**Non-productive coping**: Helplessness/Self-blame predicted a higher probability of being a cyberhate victim (OR = 1.43, CI_95%_ [1.16–1.67]), perpetrator (OR = 1.33, CI_95%_ [1.04–1.71]), and victim–perpetrator (OR = 1.91, CI_95%_ [1.52–2.36]); i.e., if participants felt helpless to cope with cyberhate, they were more likely to be victims, perpetrators, or victim–perpetrators of cyberhate than non-involved participants. Similarly, higher scores of retaliation were positively associated with higher odds of being a cyberhate victim (OR = 1.23, CI_95%_ [1.03–1.48]), perpetrator (OR = 2.27, CI_95%_ [1.81–2.86]), and victim–perpetrator (OR = 3.02, CI_95%_ [2.45–3.71]), i.e., if participants endorsed retaliation as coping strategy, they were more likely to be victims, perpetrators, or victim–perpetrators than non-involved participants.

## 4. Discussion

The present study aimed to address gaps in the literature on predictors of cyberhate involvement among adolescents. More specifically, we investigated associations between three coping styles, namely reference to others, productive coping, and non-productive coping, and being involved in cyberhate as victims, perpetrators, and victim–perpetrators, while controlling for adolescents’ age, sex, and country of origin.

We found only partial support for our first hypothesis that adolescents who endorsed *reference to others* as a coping style would show a lower risk of being involved in cyberhate. Supporting out first hypothesis, adolescents who were inclined to use distal advice as a coping strategy showed a lower risk to be cyberhate victims, perpetrators, or victim–perpetrators. However, adolescents who endorsed close support as a coping strategy did not show a lower risk of being cyberhate victims or perpetrators but showed lower odds of being victim–perpetrators of cyberhate. Our findings suggest that distal advice appeared to help reduce different forms of cyberhate involvement and that close support might be particularly important for victim–perpetrators of cyberhate. These findings are broadly in line with some prior research on the associations between reference to others and involvement as victim or perpetrator in traditional and cyber aggression [20,28,35,36,37,38,39] but in contrast to other research [30,31]. These contradicting findings stress what scholars postulated before, i.e., that there are no universally effective coping strategies and that effectiveness differs depending upon situational factors and the nature of the stressor [24].

Our second hypothesis, i.e., that adolescents who endorsed *productive coping* would show a lower risk for being involved in cyberhate, was partially confirmed as well.

Consistent with our second hypothesis, we found that adolescents who endorsed technical coping to deal with cyberhate showed consistently lower likelihoods of being cyberhate victims, perpetrators, and victim–perpetrators. This finding suggests that dealing with cyberhate by technical means (i.e., blocking the person who shares cyberhate) might be a protective coping strategy. Contradicting our second hypothesis, adolescents who endorsed assertive coping showed higher odds of being cyberhate victims. This finding is aligned with some research on cyberbullying victimization [28,29] but not with other research, which found a negative or no significant relationship between assertiveness and cybervictimization [39,48,49]. This result might indicate that showing the willingness to confront people who share cyberhate might put adolescents in danger of becoming victimized and seems not to be an appropriate coping strategy to deal with cyberhate. The available research on coping with cybervictimization shows evidence for a negative relationship between technical coping and cybervictimization [32], a positive and no significant relationship with involvement in cyberaggression as victim or perpetrator [39,48]. Other research found that *productive coping* (i.e., technical coping) is associated with higher levels of digital skills and self-efficacy [69]. We argue, therefore, that adolescents who use technical coping might be more efficacious when dealing with stressful situations and thus less likely to become involved in cyberhate. This result may be of great importance for interventions, as it could potentially explain why some adolescents prefer to not become involved in cyberhate incidents by remaining neutral observers. Thus, it appears to be important to educate adolescents to practice counter-speech without making themselves vulnerable or encourage them to use technical coping.

Finally, we found support for our third hypothesis, that unproductive coping was positively associated with cyberhate involvement. More specifically, adolescents who endorsed helplessness/self-blame or revenge as coping strategies to deal with cyberhate showed a higher likelihood to be victims, perpetrators, and victim–perpetrators. We propose that adolescents might enter a vicious circle of violence when starting to take revenge on the person who perpetrates cyberhate, while helplessness increases the risk of developing maladaptive schemas and chronification of victimization [61], thereby increasing the risk for cyberhate involvement. According to this study, cyberhate perpetration can be understood as a possible reaction to feeling helpless concerning how to deal with cyberhate constructively. This assumption is further supported by current research with students showing that adolescents perpetrate hate speech (online and offline) to compensate for feelings of frustration and inferiority [16,70]. Our findings are in line with prior research that showed a positive relationship between non-productive coping and online or offline victimization [20,30,31,46,56], as well as online and offline perpetration [31,35,56,57].

Comparing the three groups of cyberhate involvement concerning non-productive coping revealed the strongest associations for victim–perpetrators, thus verifying that adolescents who are both victims and perpetrators show the most dysfunctional psychological profiles [71]. This notion is also consistent with previous findings on cyberbullying showing that antagonistic behaviors such as retaliation are more likely amongst bully–victims than those who were victims only [72,73]. Another explanation for the more frequent use of retaliation by victim–perpetrators might be that this group tends to express more negative emotions (e.g., anger and annoyance) when confronted with stress [54].

### 4.1. Limitations and Future Directions

The present study highlights the crucial role of coping strategies for cyberhate involvement among adolescents. There are, however, a few limitations of the current investigation that need to be mentioned. First, the cross-sectional nature of the survey limits the ability to understand whether the coping strategies were antecedents or consequences of cyberhate involvement. Longitudinal studies are needed to confirm the predictive effects of coping strategies on involvement in cyberhate or vice versa. Second, cyberhate involvement was measured with single items only. Follow-up research needs to develop validated scales for measuring cyberhate involvement to avoid measurement problems associated with single-items assessments (i.e., validity, accuracy, and reliability). Third, based on a hypothetical scenario, we measured how adolescents intend to cope with cyberhate and not necessarily their actual coping behavior with experienced cyberhate victimization. Investigating adolescents’ readiness to handle cyberhate incidents can be considered an important first step in this area of research because it allows speculations about how all students—and not only those who experienced cyberhate—are likely to cope with cyberhate. Follow-up research should also consider adolescents’ actual coping behavior and the impact of cyberhate involvement. Fourth, we used a lenient cut-off value (i.e., very rarely), which allowed us to include adolescents who were occasionally involved in cyberhate. This decision was made because unlike cyberbullying, cyberhate involvement does not necessarily include a repetition. Follow-up research might compare different groups of involvement (e.g., in relation to their coping profiles) by using a lenient vs. strict cut-off score. Such research could help to understand differences among adolescents who are occasionally involved in cyberhate compared to those who are frequently involved in cyberhate. Finally, although the sample used in the present study is large and we controlled the analyses for participants’ age, sex, and country of origin, the generalizability of our findings might be limited due to the lack of a representative sample. Consequently, it would be important for future research to conduct studies based on representative samples which also allow for cross-cultural comparison.

### 4.2. Practical Implications

The findings of the present study have significant implications for the design and instruction of cyberhate prevention and intervention programs. That is, adolescents should be educated to practice distal advice and technical coping when experiencing cyberhate. The findings particularly suggest that adolescents who are not aware of an appropriate way to deal with cyberhate, or, more specifically, who feel helpless or inclined to retaliate after experiencing cyberhate should be educated in a detailed manner.

The necessity of appropriate coping skills, along with the seriousness of harm from online risks such as cyberbullying and cyberhate to adolescents, has been sufficiently emphasized [20,29,74]. However, since cyberhate is a relatively new phenomenon, not many adolescents have received media literacy education on this topic. In addition, adolescents have had few chances to practice and build the actual skills they need to deal with it. To promote adolescents to take action through professionals or via technical means, practicing coping skills might be more important. Therefore, experimental learning using online simulations, rather than a simple one-sided instruction, is essential so that adolescents can apply what they learn in real situations. For example, Social Media Test Drive, an educational tool created by Cornell University Social Media Lab [75], offers a safe and protected platform and allows adolescents to practice skills they learned using an interactive social media simulation. Education that encourages adolescents to build habits that endorse distal advice or technical coping rather than contacting people around them or cyberhate perpetrators, when adolescents experience cyberhate, can be achieved most effectively through educational technologies, which reorganize the online environment.

Research on virtual learning for coping with bullying and cyberbullying notes that participants still lack coping strategies, while the virtual programs enrich participants’ knowledge about bullying issues [76]. The authors stress the necessity of intervention designed to improve the ability to cope with the situation. Using online educational games created to address cyberhate can be an effective way. Educational games have been proved an effective tool capable of improving skills and changing behaviors as well as increasing awareness and knowledge [77,78]. A recent work by Yang et al. [47] also emphasizes online educational games about media literacy education by comparing the effects between online games and online intervention which lacks game elements. Therefore, involving game elements (e.g., scenario, feedback, and progress), which allow higher learner engagement for the intervention that helps to practice a series of coping strategies and to experience the consequences, is essential to cyberhate coping strategy training.

## 5. Conclusions

The present study is one of the first that investigated the use of coping strategies among adolescents who are involved in cyberhate as victims, perpetrators, and victim–perpetrators. The findings suggest that adolescents are using a wide range of coping strategies. That means the use of one specific type of coping strategy does not hinder the use of another. It remains to be researched whether the sum of several effective or ineffective coping strategies has a cumulative function or not. Another finding is that the use of coping strategies is related to the likelihood of being involved in cyberhate. While the readiness to use non-productive strategies (i.e., helplessness, revenge) is positively linked to involvement in cyberhate for all three roles, endorsing reference to others (i.e., distal advice) and productive coping (i.e., technical coping) showed mixed results. Our findings are particularly relevant to the scarce literature on cyberhate prevention programs and the development of educational games and virtual learning environments to counter cyberhate involvement. Such initiatives should particularly focus on educating adolescents involved in cyberhate to use distal advice and technical coping, to avoid using revenge to cope with cyberhate, and empower them so that they do not feel helpless in dealing with cyberhate.

## Figures and Tables

**Table 1:** Frequencies by Age, Sex and Country (*n* = 6722).

Age	Sex	Country																	
		Cyprus		Germany		Greece		India		South Korea		Spain		Thailand		USA		Total	
		*n*	%	*n*	%	*n*	%	*n*	%	*n*	%	*n*	%	*n*	%	*n*	%	*n*	%
12–15	Male	40	0.9	616	14.4	90	2.1	399	7.9	252	5.9	372	8.7	151	3.5	241	5.6	2101	31.3
	Female	115	2.7	625	14.6	83	1.9	294	6.9	290	6.8	387	9	162	3.8	231	5.4	2187	32.5
16–18	Male	29	1.2	120	4.9	216	8.9	278	11.4	127	5.2	119	4.9	182	7.5	140	5.8	1211	18
	Female	34	1.4	119	4.9	271	11.1	210	8.6	85	3.5	138	5.7	211	8.7	155	6.4	1223	18.2
**Total**		218	3.2	1480	22	660	9.8	1121	16.7	754	11.2	1016	15.1	706	10.5	767	11.4	6722	100

**Table 2:** Descriptive statistics and correlations (*n* = 6562).

	1	2	3	4	5	6	7	8	9	10
1. Victimization	---	0.37 ***	−0.02	−0.02	0.01	−0.05 ***	−0.07 ***	0.11 ***	0.05 ***	−0.02
2. Perpetration		---	−0.04 ***	−0.06 ***	−0.06 ***	−0.10 ***	0.04 ***	0.12 ***	0.07 ***	0.08 ***
3. Distal Advice			---	0.40 ***	0.42 ***	0.26 ***	0.36 ***	0.17 ***	−0.08 ***	−0.04 ***
4. Close Support				---	0.58 ***	0.64 ***	0.42 ***	0.33 ***	−0.04 ***	−0.14 ***
5. Assertiveness					---	0.56 ***	0.40 ***	0.26 ***	−0.02 **	−0.10 ***
6. Technical Coping						---	0.30 ***	0.26 ***	−0.03 ***	−0.12 ***
7. Helplessness							---	0.28 ***	−0.04 ***	−0.10 ***
8. Revenge								---	−0.01	0.08 ***
9. Age									---	0.01
10. Sex										---
M (SD)	0.32 (0.76)	0.22 (0.61)	1.16 (0.99)	1.77 (1.03)	1.87 (1.07)	1.82 (1.13)	0.94 (0.93)	0.93 (0.94)	14.93 (1.64)	---

** *p* < 0.01, *** *p* < 0.001.

**Table 3:** Results of Coping Strategies Predicting Involvement in Cyberhate as Victim, Perpetrator, or Victim–Perpetrator.

	Variables	Victims ^a^		Perpetrators ^a^		Victim-Perpetrators ^a^	
		Exp (B)	CI_95%_	Exp (B)	CI_95%_	Exp (B)	CI_95%_
**Reference to others**	Distal Advice	0.687 ***	0.578–0.817	0.541 ***	0.422–0.692	0.820 ***	0.726–0.925
	Close Support	1.13	0.936–1.37	1.11	0.860–1.44	0.669 **	0.533–0.840
**Productive Coping**	Assertiveness	1.23 *	1.11–1.51	0.978	0.752–1.27	0.834	0.662–1.05
	Technical Coping	0.755 **	0.622–0.918	0.533 ***	0.415–0.685	0.419 ***	0.337–0.521
**Non-Productive Coping**	Helplessness	1.43 **	1.16–1.67	1.33 *	1.04–1.71	1.91 ***	1.52–2.36
	Revenge	1.23 **	1.03–1.48	2.27 ***	1.81–2.86	3.02 ***	2.45–3.71
	**Control Variables**						
	Age	1.30 ***	1.11–1.53	1.25 ***	1.04–1.54	1.64 ***	1.36–1.98
	Being a girl ^b^	1.41 ***	1.19–1.65	0.655 ***	0.527–0.813	0.817 *	0.676–0.988
	Being German ^c^	0.352 ***	0.258–0.480	0.657	0.431–1.00	0.211 ***	0.147–0.303
	Being Greek ^c^	0.217 ***	0.147–0.321	0.502 **	0.300–0.840	0.186 ***	0.118–0.296
	Being Cypriot ^c^	0.229 ***	0.131–0.398	0.331 **	0.136–0.807	0.079 ***	0.028–0.221
	Being Spanish ^c^	0.439 ***	0.320–0.602	0.444 ***	0.276–0.716	0.139 ***	0.090–0.216
	Being Thai ^c^	0.542 ***	0.384–0.764	1.24	0.794–1.93	1.36 *	1.10–1.86
	Being Korean ^c^	0.212 ***	0.145–0.310	0.284 ***	0.160–0.503	0.064 ***	0.034–0.119
	Being Indian ^c^	0.233 ***	0.169–0.322	0.799	0.540–1.18	0.157 ***	0.110–0.224

* *p* < 0.05, ** *p* < 0.01, *** *p* < 0.001. ^a^ = reference category: non-involved, ^b^ = reference category: being a boy, ^c^ = reference category: being American.

## Data Availability

The data presented in this study are available on request from the corresponding author.

## Supplementary Materials

The following supporting information can be downloaded at: https://www.mdpi.com/article/10.3390/ijerph19116749/s1, Table S1: Coefficient Alpha of the Coping Strategy Subscales for Each Country separately; Table S2: Descriptive Statistics and Correlations among Main Study Variables for Each Country Separately; Table S3: Cyberhate Involvement by Country.
