# Supplementary material for: Associations between Coping Strategies and Cyberhate Involvement: Evidence from Adolescents across Three World Regions
Source: Int J Environ Res Public Health. 2022 May 31;19(11):6749. doi: 10.3390/ijerph19116749 (PMC9180730; doi:10.3390/ijerph19116749)
Supplement: Supplementary file 1 [file ijerph-19-06749-s001.zip › ijerph-1722644-supplementary.pdf]

International Journal of Environmental Research and Public Health

MDPI

Table S1

Coefficient Alpha of the Coping Strategy Subscales for each Country separately

Coping strategies	Cyprus	Germany	Greece	India	South Korea	Spain	Thailand	U.S.
	α	α	α	α	α	α	α	α
Distal advice	.76	.70	.73	.79	.82	.78	.91	.78
Close support	.84	.85	.83	.84	.79	.78	.92	.85
Assertiveness	.92	.91	.88	.90	,81	.86	.95	.75
Technical coping	.81	.83	.75	.78	.69	.74	.82	.72
Helplessness/ Self-blame	.88	.85	.82	.76	.69	.80	.88	.78
Retaliation	.88	.85	.87	.73	.68	.63	.85	.76

Note. Cyprus, *n* = 219; Germany, *n* = 1,418; Greece, *n* = 670; India, *n* = 1,119; South Korea, *n* = 755; Spain, *n* = 1,010; Thailand, *n* = 707; U.S., *n* = 845.

Table S2

Descriptive Statistics and Correlations among Main Study Variables for each Country separately

Country		M	SD	1.	2.	3.	4.	5.	6.	7.	8.
Cyprus											
	1. Victimization	0.16	0.56	–							
	2. Perpetration	0.05	0.28	.29**	–						
	3. Distal advice	1.40	0.97	.01	-.04	–					
	4. Close support	2.21	0.92	.04	.01	.45**	–				
	5. Assertiveness	2.05	1.05	.10*	.04	.31**	.39**	–			
	6. Technical coping	2.50	0.85	-.02	.06	.44**	.64**	.48**	–		
	7. Helplessness/ Self-blame	1.03	1.02	.18**	.09*	.40**	.33**	.27**	.27**	–	
	8. Retaliation	0.73	0.90	.24**	.17**	.14*	.11*	.27**	.17	.26**	–
Germany		M	SD	1.	2.	3.	4.	5.	6.	7.	8.
	1. Victimization	0.29	0.74	–							
	2. Perpetration	0.19	0.62	.32**	–						
	3. Distal advice	0.73	0.76	-.06*	-.09*	–					
	4. Close support	1.58	1.05	-.07**	-.12**	-.43**	–				
	5. Assertiveness	1.77	1.07	-.06*	-.14**	-32**	.57**	–			
	6. Technical coping	1.92	1.08	-.02	-.13**	.34**	-65**	.59**	–		
	7. Helplessness/ Self-blame	0.75	0.89	.03	-.01	-39**	.45**	.35**	.35**	–	
	8. Retaliation	0.76	0.94	.10**	.11**	.06*	.16**	.16**	.24**	.18**	–
Greece		M	SD	1.	2.	3.	4.	5.	6.	7.	8.
	1. Victimization	0.26	0.72	–							

	2. Perpetration	0.19	0.63	.39**	–						
	3. Distal advice	0.97	0.87	-.01	-.06	–					
	4. Close support	2.18	0.93	-.08*	-.15**	.34**	–				
	5. Assertiveness	2.06	1.01	.07	-.06	.23**	.44**				
	6. Technical coping	2.44	0.82	-.02	-.12**	.32**	.52**	.48**	–		
	7. Helplessness/ Self-blame	0.64	0.84	.05	.01	.16**	.24**	.18**	.13**	–	
	8. Retaliation	0.85	0.97	.10**	.21**	-.10**	-.09*	-.03	-.03	-.01	–
**India**		M	SD	**1.**	**2.**	**3.**	**4.**	**5.**	**6.**	**7.**	**8.**
	1. Victimization	0.25	0.71	–							
	2. Perpetration	0.24	0.63	.28**	–						
	3. Distal advice	0.84	0.88	.09**	.09**	–					
	4. Close support	1.56	1.11	.13**	.16**	.55**	–				
	5. Assertiveness	1.58	1.23	.12**	.16**	.61**	.70**	–			
	6. Technical coping	1.55	1.14	.14**	.11**	.59**	.79**	.70**	–		
	7. Helplessness/ Self-blame	1.16	1.01	.12**	.15**	.58**	.66**	.73**	.63**	–	
	8. Retaliation	1.08	1.01	.13**	.14**	.46**	.66**	.54**	.57**	.53**	–
**South Korea**		M	SD	**1.**	**2.**	**3.**	**4.**	**5.**	**6.**	**7.**	**8.**
	1. Victimization	0.14	0.49	–							
	2. Perpetration	0.07	0.36	.22**	–						
	3. Distal advice	1.61	0.95	-.07*	-.06	–					
	4. Close support	1.78	0.87	-.04	-.01	.44**	–				
	5. Assertiveness	1.97	0.87	-.03	-.05	.47**	.51**	–			
	6. Technical coping	2.17	0.82	-.06	-.03	.50**	.56**	.57**	–		
	7. Helplessness/ Self-blame	0.92	0.77	-.01	.04	.28**	.40**	.37**	.30**	–	

		M	SD	1.	2.	3.	4.	5.	6.	7.	8.
	8. Retaliation	0.99	0.79	.01	.08*	.26**	.36**	.35**	.30**	.37**	–
**Spain**		M	SD	1.	2.	3.	4.	5.	6.	7.	8.
	1. Victimization	0.28	0.69	–							
	2. Perpetration	0.12	0.49	.17**	–						
	3. Distal advice	1.36	0.96	-.14**	-.11**	–					
	4. Close support	2.26	0.84	-.08*	-.10**	.42**	–				
	5. Assertiveness	2.33	0.86	-.07*	-.13**	.29**	.48**	–			
	6. Technical coping	2.45	0.81	-.05	-.17**	.34**	.56**	.56**	–		
	7. Helplessness/ Self-blame	1.05	0.93	-.01	-.06	.26	.34	.19**	.24**	–	
	8. Retaliation	1.07	0.84	.04	.09**	.04	.18**	.25**	.19**	.16**	–
**Thailand**		M	SD	1.	2.	3.	4.	5.	6.	7.	8.
	1. Victimization	0.55	0.85	–							
	2. Perpetration	0.47	0.77	.53**	–						
	3. Distal advice	1.05	1.02	.05*	-.01	–					
	4. Close support	1.34	1.13	.14**	.02	.60**	–				
	5. Assertiveness	1.46	1.21	.14**	.02	.65**	.80**	–			
	6. Technical coping	1.25	1.09	.15**	.05	.59**	.79**	.76**	–		
	7. Helplessness/ Self-blame	0.93	1.01	.14**	.03	.59**	.69**	.61**	.64**	–	
	8. Retaliation	0.68	0.89	.22**	.19**	.36**	.38**	.36**	.45**	.45**	–
**U.S.**		M	SD	1.	2.	3.	4.	5.	6.	7.	8.
	1. Victimization	0.61	0.96	–							
	2. Perpetration	0.34	0.70	.45**	–						
	3. Distal advice	1.83	1.05	-.15**	-.10**	–					
	4. Close support	1.65	0.86	.01	.01	.06	–				

5. Assertiveness	1.87	0.91	-.04	-.03	.41**	.14**	–			
6. Technical coping	0.68	0.84	.10*	.10**	-.03	.35**	.01	–		
7. Helplessness/ Self-blame	1.05	0.90	.08*	.09*	.16**	.21**	.37**	.24**	–	
8. Retaliation	1.09	0.99	.13**	.11**	-.10**	.61**	.01	.40**	.09**	–

Note. Cyprus, *n* = 210; Germany, *n* = 1,385; Greece, *n* = 659; India, *n* = 1,118; South Korea, *n* = 745; Spain, *n* = 993; Thailand, *n* = 607; U.S., *n* = 845. ** Correlation is significant at the 0.01 level (2-tailed). * Correlation is significant at the 0.05 level (2-tailed).

Table S3

Cyberhate Involvement by Country

**Cyberhate Involvement**	Cyprus		Germany		Greece		India		South Korea		Spain		Thailand		U.S.	
	%	n	%	n	%	n	%	n	%	n	%	n	%	n	%	n
**Victim**	8.3	18	11.7	165	9.7	65	8.4	94	7	53	14.2	143	12.4	88	18.2	154
**Perpetrator**	2.8	6	6	85	5.8	39	9.5	106	2.6	20	4.1	41	8.2	58	6.4	54
**Victim-Perpetrator**	1.8	4	5.3	75	5.2	35	5.7	64	1.6	12	3.5	35	24	170	18.1	153
**Non-involved**	87.1	189	77	1090	79.2	530	76.4	854	88.7	670	78.3	788	55.4	393	57.3	484

Note. Cyprus, *n* = 217; Germany, *n* = 1,415; Greece, *n* = 669; India, *n* = 1,118; South Korea, *n* = 755; Spain, *n* = 1,007; Thailand, *n* = 709; U.S., *n* = 845.
